# The dichotomy of central nervous system cancer burden: A global analysis revealing diagnosis-driven epidemics and therapy-resistant mortality

**DOI:** 10.1093/noajnl/vdag171

**Published:** 2026-07-09

**Authors:** Lanlan Guo, Haoyue Hu, Mingying Xiao, Wei Chen, Yuanyang Huang, Xuan Li, Ziwei Wu, Suixian Zhang, Ying Wang, Baiqiang Dong, Chenfei Wu, Yonggao Mou, Yuanyuan Chen

**Affiliations:** Department of Radiation Oncology, Sun Yat-sen University Cancer Center, Guangzhou, P. R. China; State Key Laboratory of Oncology in South China, Guangzhou, P. R. China; Collaborative Innovation Center for Cancer Medicine, Guangzhou, P. R. China; Guangdong Key Laboratory of Nasopharyngeal Carcinoma Diagnosis and Therapy, Guangzhou, P. R. China; State Key Laboratory of Oncology in South China, Collaborative Innovation Center for Cancer Medicine, Sun Yat-sen University Cancer Center, Guangzhou, P. R. China; Department of Radiation Oncology, Sun Yat-sen University Cancer Center, Guangzhou, P. R. China; State Key Laboratory of Oncology in South China, Guangzhou, P. R. China; Collaborative Innovation Center for Cancer Medicine, Guangzhou, P. R. China; Guangdong Key Laboratory of Nasopharyngeal Carcinoma Diagnosis and Therapy, Guangzhou, P. R. China; State Key Laboratory of Oncology in South China, Collaborative Innovation Center for Cancer Medicine, Sun Yat-sen University Cancer Center, Guangzhou, P. R. China; Department of Radiation Oncology, Sun Yat-sen University Cancer Center, Guangzhou, P. R. China; State Key Laboratory of Oncology in South China, Guangzhou, P. R. China; Collaborative Innovation Center for Cancer Medicine, Guangzhou, P. R. China; Guangdong Key Laboratory of Nasopharyngeal Carcinoma Diagnosis and Therapy, Guangzhou, P. R. China; State Key Laboratory of Oncology in South China, Collaborative Innovation Center for Cancer Medicine, Sun Yat-sen University Cancer Center, Guangzhou, P. R. China; Department of Radiation Oncology, Sun Yat-sen University Cancer Center, Guangzhou, P. R. China; State Key Laboratory of Oncology in South China, Guangzhou, P. R. China; Collaborative Innovation Center for Cancer Medicine, Guangzhou, P. R. China; Guangdong Key Laboratory of Nasopharyngeal Carcinoma Diagnosis and Therapy, Guangzhou, P. R. China; State Key Laboratory of Oncology in South China, Collaborative Innovation Center for Cancer Medicine, Sun Yat-sen University Cancer Center, Guangzhou, P. R. China; Department of Radiation Oncology, Sun Yat-sen University Cancer Center, Guangzhou, P. R. China; State Key Laboratory of Oncology in South China, Guangzhou, P. R. China; Collaborative Innovation Center for Cancer Medicine, Guangzhou, P. R. China; Guangdong Key Laboratory of Nasopharyngeal Carcinoma Diagnosis and Therapy, Guangzhou, P. R. China; State Key Laboratory of Oncology in South China, Collaborative Innovation Center for Cancer Medicine, Sun Yat-sen University Cancer Center, Guangzhou, P. R. China; Department of Radiation Oncology, Sun Yat-sen University Cancer Center, Guangzhou, P. R. China; State Key Laboratory of Oncology in South China, Guangzhou, P. R. China; Collaborative Innovation Center for Cancer Medicine, Guangzhou, P. R. China; Guangdong Key Laboratory of Nasopharyngeal Carcinoma Diagnosis and Therapy, Guangzhou, P. R. China; State Key Laboratory of Oncology in South China, Collaborative Innovation Center for Cancer Medicine, Sun Yat-sen University Cancer Center, Guangzhou, P. R. China; Department of Radiation Oncology, Sun Yat-sen University Cancer Center, Guangzhou, P. R. China; State Key Laboratory of Oncology in South China, Guangzhou, P. R. China; Collaborative Innovation Center for Cancer Medicine, Guangzhou, P. R. China; Guangdong Key Laboratory of Nasopharyngeal Carcinoma Diagnosis and Therapy, Guangzhou, P. R. China; State Key Laboratory of Oncology in South China, Collaborative Innovation Center for Cancer Medicine, Sun Yat-sen University Cancer Center, Guangzhou, P. R. China; Department of Radiation Oncology, Sun Yat-sen University Cancer Center, Guangzhou, P. R. China; State Key Laboratory of Oncology in South China, Guangzhou, P. R. China; Collaborative Innovation Center for Cancer Medicine, Guangzhou, P. R. China; Guangdong Key Laboratory of Nasopharyngeal Carcinoma Diagnosis and Therapy, Guangzhou, P. R. China; State Key Laboratory of Oncology in South China, Collaborative Innovation Center for Cancer Medicine, Sun Yat-sen University Cancer Center, Guangzhou, P. R. China; Department of Radiation Oncology, Sun Yat-sen University Cancer Center, Guangzhou, P. R. China; State Key Laboratory of Oncology in South China, Guangzhou, P. R. China; Collaborative Innovation Center for Cancer Medicine, Guangzhou, P. R. China; Guangdong Key Laboratory of Nasopharyngeal Carcinoma Diagnosis and Therapy, Guangzhou, P. R. China; State Key Laboratory of Oncology in South China, Collaborative Innovation Center for Cancer Medicine, Sun Yat-sen University Cancer Center, Guangzhou, P. R. China; Department of Radiation Oncology, Sun Yat-sen University Cancer Center, Guangzhou, P. R. China; State Key Laboratory of Oncology in South China, Guangzhou, P. R. China; Collaborative Innovation Center for Cancer Medicine, Guangzhou, P. R. China; Guangdong Key Laboratory of Nasopharyngeal Carcinoma Diagnosis and Therapy, Guangzhou, P. R. China; State Key Laboratory of Oncology in South China, Collaborative Innovation Center for Cancer Medicine, Sun Yat-sen University Cancer Center, Guangzhou, P. R. China; Department of Radiation Oncology, Sun Yat-sen University Cancer Center, Guangzhou, P. R. China; State Key Laboratory of Oncology in South China, Guangzhou, P. R. China; Collaborative Innovation Center for Cancer Medicine, Guangzhou, P. R. China; Guangdong Key Laboratory of Nasopharyngeal Carcinoma Diagnosis and Therapy, Guangzhou, P. R. China; State Key Laboratory of Oncology in South China, Collaborative Innovation Center for Cancer Medicine, Sun Yat-sen University Cancer Center, Guangzhou, P. R. China; Department of Radiation Oncology, Sun Yat-sen University Cancer Center, Guangzhou, P. R. China; State Key Laboratory of Oncology in South China, Guangzhou, P. R. China; Collaborative Innovation Center for Cancer Medicine, Guangzhou, P. R. China; Guangdong Key Laboratory of Nasopharyngeal Carcinoma Diagnosis and Therapy, Guangzhou, P. R. China; State Key Laboratory of Oncology in South China, Collaborative Innovation Center for Cancer Medicine, Sun Yat-sen University Cancer Center, Guangzhou, P. R. China; Department of Radiation Oncology, Sun Yat-sen University Cancer Center, Guangzhou, P. R. China; State Key Laboratory of Oncology in South China, Guangzhou, P. R. China; Collaborative Innovation Center for Cancer Medicine, Guangzhou, P. R. China; Guangdong Key Laboratory of Nasopharyngeal Carcinoma Diagnosis and Therapy, Guangzhou, P. R. China; State Key Laboratory of Oncology in South China, Collaborative Innovation Center for Cancer Medicine, Sun Yat-sen University Cancer Center, Guangzhou, P. R. China

**Keywords:** central nervous system cancer, epidemiology, glioblastoma, global burden of disease, therapy resistance

## Abstract

**Background:**

The global burden of central nervous system (CNS) cancers is rising unevenly. Existing studies lack a framework linking macro trends to underlying drivers across scales.

**Methods:**

We constructed a “macro-meso-micro” framework, integrating Global Burden of Disease 2021 data (macro perspective) and US SEER registry data (meso perspective). Global/regional/national incidence and mortality trends (1990-2021) were analyzed using descriptive statistics and Bayesian models, alongside subtype-specific clinical patterns.

**Results:**

The global age-standardized incidence rate (ASIR) increased from 3.82 to 4.28 per 100,000 population (1990-2021), while the age-standardized mortality rate (ASMR) declined slowly (3.08-3.06), revealing a core “incidence-mortality decoupling.” Incidence correlated positively with the socio-demographic index (SDI), but some high-SDI regions achieved superior mortality control, indicating healthcare disparities. SEER data clarified that this plateau was driven by the dismal survival of aggressive subtypes like glioblastoma (GBM), contrasting sharply with the rising detection of benign/low-grade tumors (eg, meningioma). For GBM, standard care has reached a low therapeutic ceiling, with most patients relapsing into a treatment void.

**Conclusions:**

CNS cancer burden evolution resulted from multi-scale diagnostic, therapeutic, and biological interactions. Our framework connected macro trends, meso subtype heterogeneity, and micro resistance mechanisms, pinpointing the conquest of therapy-resistant, high-grade tumors (especially GBM) as the central challenge. A translational pathway of “macro surveillance for target identification—clinical problem definition—mechanistic research for breakthrough” was proposed to guide future intervention.

Key Points

- We pioneer a macro-meso-micro framework, revealing the CNS cancer burden paradox: rising incidence (diagnosis-driven) vs. plateaued mortality (therapy-resistant GBM).
- This pinpoints GBM’s therapeutic ceiling as the key factor and charts a translational roadmap.

Importance of the Study

The significance of this study extends beyond conventional burden description, primarily in three aspects:

1. Innovation in Theoretical Framework: It provides the first multi-scale analytical paradigm capable of coherently explaining patterns from global inequalities and subtype heterogeneity down to cellular mechanisms, offering a novel conceptual tool for neuro-oncology epidemiology.
2. Challenging Conventions and Refocusing Priorities: The study confirms that the rise in overall incidence primarily reflects improved diagnostic capacity, not a true surge in malignant disease. This finding compelled the academic community, funders, and policymakers to refocus core resources and attention on the fundamental challenge of overcoming therapy resistance in high-grade malignancies.
3. Informing Translation and Policy: The research translates abstract burden data into a concrete, actionable scientific and clinical agenda. It provides an evidence base for optimizing global healthcare resource allocation (eg, strengthening diagnostic capabilities in high-burden regions while supporting innovative therapy development for GBM worldwide) and sets clear priority targets for translational research from basic science to clinical practice.

Brain and central nervous system cancer (collectively referred to as CNS cancers) refers to cancers that occur in the brain and the central nervous system. The central nervous system includes the brain, spinal cord, and optic nerves, and so on. The cancers can be primary, meaning they originate from the tissues of the central nervous system, such as brain tumors; or secondary, which means they have metastasized to the brain from cancers in other sites of the body, like lung or breast. Here, when it comes to CNS cancers, we refer to the primary ones.

These cancers can affect different parts of the brain, including the cerebral cortex (principally for advanced functions, such as thought, cognition, and language), the brainstem (principally for functions of breath and heartbeat), and the cerebellum (principally for coordinating movement and balance). The symptoms and severity of CNS cancers depend on the size, location, and growth rate of the tumor, as well as whether it would cause pressure on adjacent tissues. The treatment methods for these cancers include surgery, radiation therapy, chemotherapy, targeted therapy, and so forth. The treatment strategy needs to be determined based on the type, stage of the cancer, and general condition of patients.

CNS cancers include a variety of tumor types, the 5-year survival rate for which was estimated to be around 34.8%.^1^ Brain tumors account for the majority (85%-90%) of all CNS cancers.^2^ Additionally, according to GLOBOCAN, brain tumors rank as the 19th most common type of cancer worldwide.^3^ Additionally, around 180,000 deaths from brain tumors are reported annually, which takes up 2.03% of all cancer-related deaths worldwide.^4^ Compared to 2000, the 5-year (36%) and 10-year (31%) survival rates were higher in 2020.^2^ Even with advancements in treatment approaches for patients with brain tumors, their survival rates remain substantially lower than those of many other common malignancies.^5-11^

The incidence of different histology varies greatly, with gliomas (including glioblastoma, astrocytomas, oligodendrogliomas, and ependymal tumors) accounting for the largest proportion of CNS cancers.^1^ Moreover, of gliomas, the astrocytic tumor group was the most common histological type, followed by oligodendrogliomas, other mixed gliomas, and malignant meningiomas.^12^ CNS cancers remain the second most common type among individuals aged 15-39, defined as adolescents and young adults (AYA) by the Central Brain Tumor Registry of the United States, with an incidence rate of 12.21 per 100,000 population.^1^ In recent years, overall cancer survival rates have improved for this population.^13^ The management of AYA patients with CNS cancers is particularly challenging due to the lack of standardized treatment guidelines, as clinicians must decide whether to follow pediatric protocols or adult protocols (generally less aggressive). This “care gap” may partly explain the relatively slower survival improvement and lower clinical trial enrollment observed in this population.

With advancements in treatment over the past years, the 5-year survival rate for pediatric patients with CNS cancers has increased from 50% to 80% in selected subtypes, although the magnitude of improvement varies considerably by tumor type.^14^^,^^15^ However, survival often comes at the cost of sacrificing the health and well-being of survivors. Nevertheless, the mortality rate for AYAs with CNS cancers increased by 0.6% annually from 2007 to 2016, especially with a 1% increase per year for females.^16^ The AYA age group, which is at the intersection of pediatric and adult healthcare, is a distinct patient cohort that is not well-researched. Indeed, the low enrollment of AYA individuals in clinical trials has been correlated with worse survival outcomes.^16^

Therefore, CNS cancers are a vital source of cancer-related incidence and mortality worldwide. Regional differences in incidence may provide clues to genetic or environmental causes, along with a foundation for broadening epidemiological knowledge. A comprehensive understanding of the epidemiology of CNS cancers is crucial for researchers, public health officials, disease advocacy groups, and clinicians, and promotes fostering collaboration for future research. In this context, it is of great significance to understand the distribution of CNS cancers, which facilitates local, national, and international efforts to appropriately allocate healthcare resources.

However, while the Global Burden of Diseases (GBD) studies provide a global perspective, their classification of tumor subtypes is coarse, lacking distinction between benign and malignant or high- and low-grade entities. Research based on high-quality registries exists, such as the US Surveillance, Epidemiology, and End Results (SEER) database, which we used in this study. The database offers detailed subtype data but is predominantly regional or national in scope, lacking global representativeness and analyses of inequalities. Furthermore, existing studies often remain at the level of trend description, failing to delve into the underlying driving forces behind these trends. Based on these limitations, the field of central nervous system tumors lacks a systematic analytical framework capable of integrating “macro-global trends” with “meso-subtype profiling” and further down to “micro-mechanistic insights.” In this study, we integrated GBD (global perspective) and SEER (high-resolution subtype data) to conduct a cross-scale, complementary analysis. This approach aimed to further quantify the contributions of diagnosis and treatment to burden trends and to explain and predict the evolution of the CNS cancer burden.

## Methods

### Data Source

GBD 2021 comprehensively assessed the latest epidemiological data and improved standardized methods to comprehensively assess mortality and incidence rates, 369 diseases and injuries, and 87 risk factors in 204 countries and regions from 1990 to 2021. It is worth noting that the indicators in GBD 2021 include the estimated values with 95% uncertainty intervals (UIs). According to the GBD algorithm, these are defined by the 2.5th and 97.5th ordered values from a sample of 1,000 draws from the posterior distribution, with all incidence rates reported per 100,000 population. To obtain the data, the study employed the Global Health Data Exchange (GHDx) query tool (http://ghdx.healthdata.org/gbd-results-tool), which is maintained by the Institute for Health Metrics and Evaluation. We utilized the GBD 2021 dataset to analyze trends and patterns in the disease burden of CNS cancers, extracting data on the incidence, mortality, age-standardized incidence rates (ASIR), and age-standardized mortality rates (ASMR) from 1990 to 2021. Furthermore, the analysis considered parameters such as sex, age, and different demographic and geographic factors, providing valuable insights into the global epidemiology of CNS cancers over a particular period. Herein, “brain and central nervous system cancer” was selected as the cause; “female,” “male” and “both” for the sex; and “incidence,” “death” for the key metrics from the database.

### Patients

This study integrated two databases to address their respective limitations. GBD data were used to analyze the global burden of CNS cancers. According to the official definition of the GBD 2021 study, brain and CNS cancer includes all cancers coded as C70.0-C72.9 (C70, malignant neoplasm of meninges; C71, malignant neoplasm of brain; C72, malignant neoplasm of spinal cord, cranial nerves and other parts of central nervous system). Thus, the GBD analysis covers only malignant CNS cancers, excluding benign or borderline tumors. The SEER database was employed for high-resolution subtype-specific analyses. Based on the WHO classification of CNS cancers, we selected two representative subtypes for comparative analysis: meningioma (as a prototype of benign/indolent tumors) and glioblastoma (as the most aggressive malignant tumor). The SEER database^17^ is a population-based cancer registry system managed by the National Cancer Institute, covering approximately 28% of the US. The SEER database offers a pathologically based, highly refined classification of tumor subtypes, making it an ideal model for elucidating how diagnostic techniques and evolving disease classifications influence incidence trends. This analysis aimed to leverage this high-quality data to provide potential biological and clinical mechanistic explanations for the overall trends observed in the GBD study. Patient data were derived from the database. According to the AJCC 8th edition criteria, this study included patients with histologically confirmed glioblastoma, embryonal tumors, and meningioma between 2000 and 2020. Extracted variables included age, sex, race, survival status, cause of death, and other relevant information. In this study, “sex” refers to biological sex (male/female).

### Estimation Methods

For CNS cancers, the GBD 2021 study utilized the International Classification of Diseases, eleventh revision (ICD-11) codes as 2A00 to 2A0Z (2A00, primary neoplasms of brain; 2A01, primary neoplasms of meninges; 2A02, primary neoplasm of spinal cord, cranial nerves or remaining parts of central nervous system; 2A0Z, other and unspecified neoplasms of brain or central nervous system). Previous GBD publications provided detailed information on the methods used for estimating disease burden, such as incidence, mortality, prevalence, years lived with disability (YLDs), years of life lost (YLLs), and disability-adjusted life-years (DALYs).^18-20^ In short, global and regional statistical models for death and disability were constructed using cause-of-death data from death databases and data extracted from systematic reviews. The Cause of Death Ensemble Model approach was employed to determine mortality rates and the total death count. Age-standardized incidence rates (ASIR) and age-standardized mortality rates (ASMR) were standardized to the GBD world population and recorded as the numbers per 100,000 population.^21^

### Statistical Analysis

We obtained the annual incidence, deaths, DALYs, incidence, and mortality rates for CNS cancers from 1990 to 2021, which were reported in the study, accompanied by 95% UIs. The 95% UIs were used to reduce the impact of heterogeneity due to sampling error and non-sampling variance. This method provides a range of values that reflect the uncertainty in statistical modeling, offering a more comprehensive understanding of the variability in the data. Additionally, the global, regional, and national burden of CNS cancers was represented by age-standardized rates (ASRs; per 100,000 population) calculated using the world standard population to ensure comparability across different populations and periods from the GBD 2021 study, which included ASIR and ASMR. To evaluate the temporal trends, we determined the annual percentage change (APC) along with the corresponding 95% confidence interval (CI). The temporal trends in the burden of CNS cancers from 1990 to 2021 were examined by applying the estimated annual percentage change (EAPC) calculated based on ASRs from the Joinpoint regression program (version 5.1.0, April 2024), developed by the Statistical Research and Applications Branch at the National Cancer Institute in Bethesda. The GBD 2021 data used in this study were systematically adjusted for the COVID-19 pandemic period (2020-2021) within its modeling framework; therefore, Joinpoint regression analysis was performed on the complete 1990-2021 time series without excluding any calendar years. Numerous methods were employed to calculate and forecast the burden of diseases. Herein, the future trends of the CNS cancer burden were predicted using a Bayesian APC analysis (BAPC) with integrated nested Laplace approximations. BAPC was demonstrated to have better coverage and accuracy compared to other forecasting methods, as shown in previous studies.^22-24^  *P <* .05 was considered statistically significant. All statistical analyses and visualizations were performed with R Software (version 4.5.1). The study adhered to the STROBE statement, and the results were presented according to the PRISMA statement.^25^

## Results

### Macro-Level Perspective: Unequal Distribution of Global Burden of CNS Cancers

#### The long-term decoupling between incidence and mortality rates

**Figure 1.**
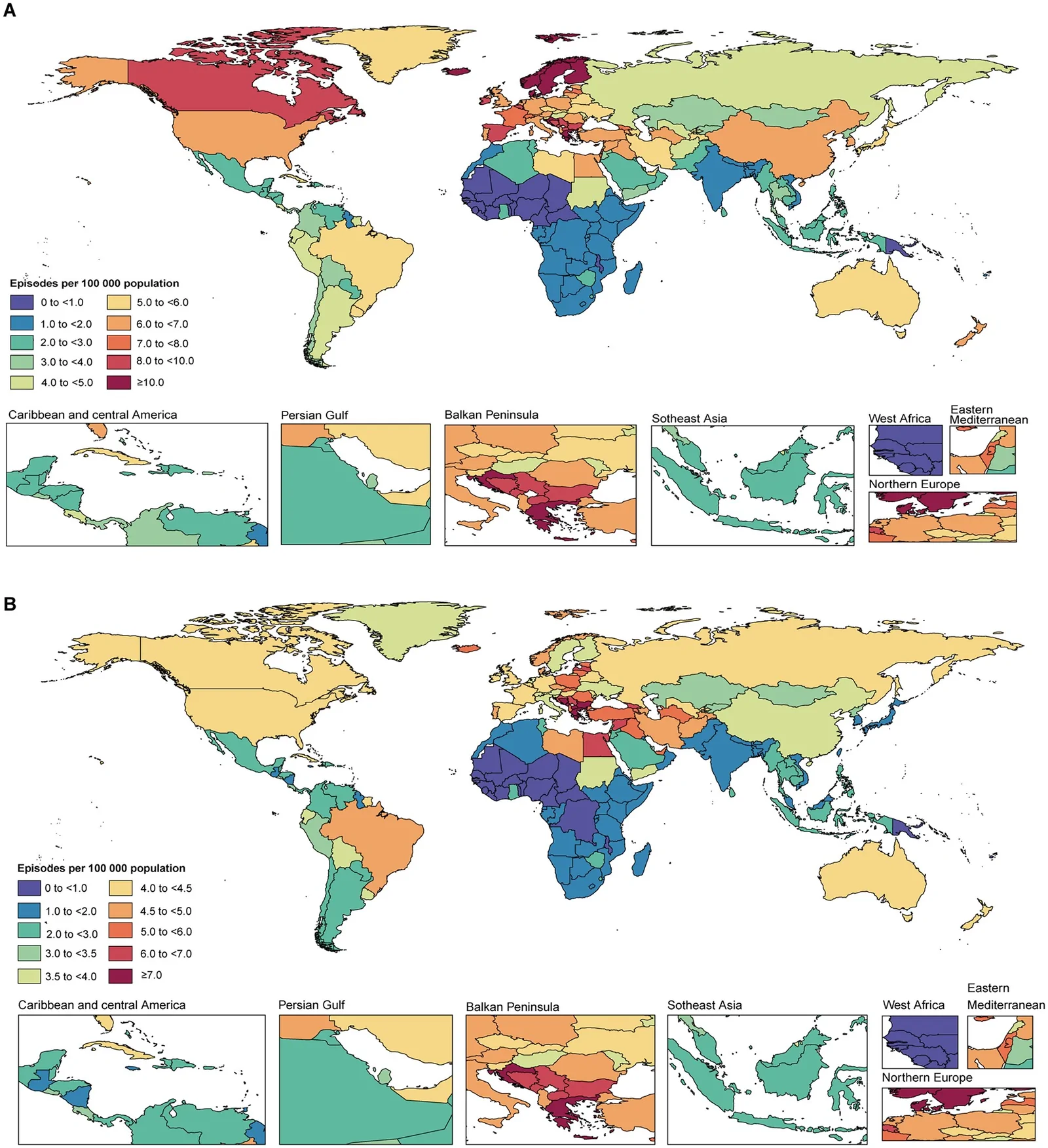
Spatial distribution of the appendicitis burden at all ages and in both sexes, 2021. CNS cancers = brain and central nervous system cancer; ASIR = age-standardized incidence rates; ASMR = age-standardized mortality rates. (A) ASIR of CNS cancers in 2021, per 100,000 population. (B) ASMR of CNS cancers in 2021, per 100,000 population.

At the nation level, the ASIR of CNS cancers per 100,000 population in 2021 was highest in Kingdom of Norway (14.96; 95% UI, 13.93 to 16.07), followed by Kingdom of Denmark (14.17; 95% UI, 12.90 to 15.42) and Principality of Monaco (13.91; 95% UI, 9.62 to 18.37) (Figure 1A). The ASMR of CNS cancers per 100,000 population in 2021 was highest in Montenegro (7.77; 95% UI 6.11 to 10.25), followed by North Macedonia (7.55; 95% UI, 5.14 to 9.98) and the Republic of Bulgaria (7.46; 95% UI, 6.21 to 8.94) (Figure 1B).

In 1990, we estimated 173,086 (95% UI, 147,452 to 194,951) cases of CNS cancers globally (Table 1). Globally, in 2021, we estimated 357,482 (95% UI, 310,457 to 407,433) cases, which was a significant increase compared to the year 1990. From 1990 to 2021, the analysis revealed a key paradox that the global age-standardized incidence rate (ASIR) of CNS cancers increased from 3.82 to 4.28 per 100,000 population, while the age-standardized mortality rate (ASMR) only slightly decreased from 3.08 to 3.06 per 100,000 population during the same period (Table 2). This pattern of significant increase in incidence versus minimal change in mortality strongly suggests that global improvements in diagnostic capacity leading to more cases being detected, while coexisting with insufficient treatment effectiveness for aggressive subtypes, resulting in eventual patient mortality.

**Table 1.** Brain and central nervous system cancer incidence, ASIR for all ages in 1990 and 2021, and incidence rate percentage change from 1990 to 2021, globally and by SDI quintile, GBD super-region, and GBD regions

Location	1990		2021		Incidence rate change between 1990 and 2021 (95% UI)
	Incidence counts (95% UI)	ASIR (95% UI)	Incidence counts (95% UI)	ASIR (95% UI)	
Global	173,086 (147,452 to 194,951)	3.82 (3.34 to 5.00)	357,482 (310,457 to 407,433)	4.28 (3.71 to 4.88)	0.4 (0.24 to 0.55)
High SDI	49,764 (44,199 to 62,918)	5.39 (4.79 to 6.81)	100,994 (94,964 to 105,629)	6.38 (6.08 to 6.64)	0.46 (0.4 to 0.51)
High-middle SDI	51,363 (45,198 to 61,117)	4.60 (4.06 to 5.51)	97,818 (83,423 to 112,505)	5.9 (5.05 to 6.77)	0.61 (0.4 to 0.84)
Middle SDI	47,003 (37,644 to 64,202)	3.28 (2.72 to 4.42)	106,918 (86,956 to 128,833)	4.11 (3.34 to 4.94)	0.6 (0.32 to 0.93)
Low-middle SDI	22,556 (16,512 to 36,947)	2.30 (1.83 to 3.55)	39,604 (31,858 to 49,414)	2.34 (1.89 to 2.93)	0.51 (0.16 to 0.83)
Low SDI	8298 (5283 to 14,554)	1.78 (1.27 to 2.80)	11,810 (8190 to 15,144)	1.43 (1 to 1.82)	0.13 (−0.17 to 0.38)
Central Europe, Eastern Europe, and Central Asia	19,627 (18,115 to 25,051)	4.37 (4.04 to 5.60)	30,649 (28,878 to 32,702)	5.59 (5.26 to 5.96)	0.62 (0.49 to 0.76)
High-income	58,431 (51,346 to 73,069)	5.62 (4.93 to 7.03)	107,967 (101,294 to 112,237)	6.69 (6.39 to 6.92)	0.47 (0.4 to 0.51)
Latin America and Caribbean	8556 (7486 to 12,768)	2.68 (2.36 to 3.99)	26,452 (24,650 to 28,436)	4.29 (3.99 to 4.63)	0.82 (0.69 to 0.96)
North Africa and Middle East	10,901 (7256 to 16,332)	4.08 (2.95 to 5.80)	26,566 (19,734 to 32,479)	4.98 (3.7 to 6.1)	0.42 (−0.05 to 0.72)
South Asia	19,935 (14,074 to 30,847)	2.04 (1.55 to 3.00)	31,817 (26,009 to 43,157)	1.87 (1.53 to 2.55)	0.35 (0.02 to 0.97)
Southeast Asia, East Asia, and Oceania	55,731 (42,421 to 74,961)	3.83 (3.00 to 5.10)	124,387 (96,845 to 154,893)	4.91 (3.81 to 6.04)	0.73 (0.38 to 1.16)
Sub-Saharan Africa	5866 (3856 to 9593)	1.43 (1.06 to 2.04)	9645 (6437 to 11,985)	1.16 (0.77 to 1.41)	0.2 (−0.2 to 0.43)
Andean Latin America	838 (665 to 1161)	2.76 (2.2 to 3.73)	2794 (2222 to 3479)	4.43 (3.52 to 5.5)	0.91 (0.39 to 1.54)
Australasia	1393 (1343 to 1448)	6.32 (6.1 to 6.56)	2536 (2342 to 2730)	5.95 (5.54 to 6.38)	0.19 (0.11 to 0.29)
Caribbean	897 (824 to 1131)	2.93 (2.72 to 3.61)	1971 (1725 to 2270)	3.85 (3.37 to 4.46)	0.63 (0.41 to 0.85)
Central Asia	1966 (1640 to 2244)	3.24 (2.68 to 3.69)	4636 (4013 to 5300)	4.94 (4.29 to 5.62)	0.71 (0.39 to 1.11)
Central Europe	8003 (7657 to 8532)	5.76 (5.5 to 6.15)	11,721 (10,689 to 12,787)	6.64 (6.07 to 7.25)	0.59 (0.46 to 0.71)
Central Latin America	2825 (2740 to 2916)	2.23 (2.17 to 2.29)	7586 (6775 to 8533)	2.98 (2.66 to 3.36)	0.74 (0.55 to 0.96)
Central Sub-Saharan Africa	295 (213 to 419)	0.83 (0.6 to 1.07)	876 (586 to 1182)	1.03 (0.67 to 1.39)	0.19 (−0.23 to 0.6)
East Asia	48,578 (35,372 to 60,360)	4.63 (3.39 to 5.77)	107,614 (83,225 to 135,768)	6.02 (4.69 to 7.53)	0.83 (0.44 to 1.33)
Eastern Europe	9101 (8400 to 9678)	3.63 (3.37 to 3.85)	14,293 (13,134 to 15,494)	4.98 (4.6 to 5.38)	0.72 (0.52 to 0.97)
Eastern Sub-Saharan Africa	1848 (1234 to 2725)	1.25 (0.81 to 1.67)	4821 (3457 to 6278)	1.53 (1.05 to 1.92)	0.17 (−0.23 to 0.48)
High-income Asia Pacific	5649 (5037 to 5978)	3.11 (2.76 to 3.29)	16,368 (14,108 to 18,001)	5.44 (4.76 to 5.92)	1.71 (1.42 to 1.95)
High-income North America	22,170 (21,558 to 22,637)	7.11 (6.93 to 7.24)	36,462 (34,376 to 37,686)	7.08 (6.75 to 7.31)	0.25 (0.21 to 0.29)
Oceania	32 (16 to 43)	0.69 (0.35 to 0.94)	82 (43 to 110)	0.74 (0.38 to 0.99)	0.19 (−0.02 to 0.44)
Southeast Asia	7051 (5029 to 8699)	1.97 (1.43 to 2.45)	16,691 (12,170 to 19,950)	2.4 (1.76 to 2.87)	0.58 (0.22 to 0.9)
Southern Latin America	1485 (1350 to 1622)	3.12 (2.84 to 3.41)	2981 (2776 to 3173)	3.81 (3.55 to 4.06)	0.47 (0.29 to 0.66)
Southern Sub-Saharan Africa	569 (441 to 729)	1.57 (1.2 to 1.98)	1390 (1024 to 1668)	2.05 (1.5 to 2.43)	0.59 (0.34 to 0.86)
Tropical Latin America	4967 (4716 to 5251)	4.14 (3.93 to 4.4)	14,101 (13,434 to 14,673)	5.65 (5.37 to 5.88)	0.9 (0.78 to 1.02)
Western Europe	30,521 (29,740 to 31,118)	6.55 (6.4 to 6.66)	49,619 (46,606 to 51,779)	7.44 (7.13 to 7.71)	0.43 (0.37 to 0.48)
Western Sub-Saharan Africa	762 (515 to 997)	0.43 (0.3 to 0.53)	2558 (1315 to 3276)	0.63 (0.34 to 0.79)	0.32 (−0.2 to 0.67)

For comparisons of incidence or mortality rates between two time points, the difference is considered statistically significant if the two 95% UIs do not overlap.

Abbreviations: ASIR, age-standardized incidence rates; GBD, Global Burden of Diseases, Injuries, and Risk Factors Study; SDI, socio-demographic index; UI, uncertainty intervals.

**Table 2.** Brain and central nervous system cancer incidence, ASMR for all ages in 1990 and 2021, and mortality rate percentage change from 1990 to 2021, globally and by SDI quintile, GBD super-region, and GBD regions

Location	1990		2021		Mortality rate change between 1990 and 2021 (95% UI)
	Death counts (95% UI)	ASMR (95% UI)	Death counts (95% UI)	ASMR (95% UI)	
Global	139,632 (119,904 to 182,291)	3.08 (2.71 to 4.00)	258,627 (222,185 to 296,134)	3.06 (2.62 to 3.5)	0.28 (0.13 to 0.44)
High SDI	34,069 (30,412 to 42,767)	3.54 (3.16 to 4.45)	63,268 (59,449 to 66,058)	3.54 (3.36 to 3.68)	0.35 (0.29 to 0.39)
High-middle SDI	42,083 (37,243 to 49,826)	3.80 (3.37 to 4.52)	71,070 (60,266 to 81,226)	3.94 (3.36 to 4.51)	0.43 (0.25 to 0.64)
Middle SDI	38,405 (30,911 to 51,842)	2.89 (2.39 to 3.82)	79,779 (64,859 to 96,617)	3.02 (2.45 to 3.65)	0.42 (0.19 to 0.71)
Low-middle SDI	18,371 (13,848 to 29,471)	2.05 (1.66 to 3.09)	33,996 (27,308 to 42,717)	2.08 (1.68 to 2.62)	0.46 (0.14 to 0.77)
Low SDI	6653 (4329 to 11,380)	1.60 (1.17 to 2.42)	10,225 (7080 to 13,086)	1.33 (0.93 to 1.69)	0.08 (−0.21 to 0.31)
Central Europe, Eastern Europe, and Central Asia	17,191 (15,956 to 22,133)	3.78 (3.51 to 4.87)	27,489 (25,917 to 29,211)	4.76 (4.49 to 5.07)	0.65 (0.53 to 0.78)
High-income	40,182 (35,402 to 50,007)	3.68 (3.24 to 4.56)	66,575 (62,329 to 69,083)	3.63 (3.46 to 3.74)	0.35 (0.3 to 0.39)
Latin America and Caribbean	6858 (6005 to 10,175)	2.33 (2.04 to 3.46)	22,101 (20,599 to 23,670)	3.58 (3.33 to 3.84)	0.82 (0.69 to 0.95)
North Africa and Middle East	8404 (5858 to 12,350)	3.55 (2.59 to 4.93)	22,280 (16,570 to 27,228)	4.44 (3.31 to 5.43)	0.39 (−0.05 to 0.69)
South Asia	16,209 (11,622 to 24,770)	1.81 (1.41 to 2.60)	27,080 (22,307 to 37,097)	1.63 (1.35 to 2.24)	0.28 (−0.02 to 0.87)
Southeast Asia, East Asia, and Oceania	46,057 (35,183 to 61,209)	3.36 (2.64 to 4.41)	84,852 (64,654 to 106,290)	3.19 (2.42 to 3.95)	0.42 (0.12 to 0.79)
Sub-Saharan Africa	4728 (3244 to 7602)	1.30 (0.99 to 1.79)	8250 (5451 to 10,250)	1.07 (0.7 to 1.3)	0.16 (−0.22 to 0.38)
Andean Latin America	721 (573 to 986)	2.51 (2 to 3.36)	2199 (1751 to 2725)	3.55 (2.83 to 4.4)	0.75 (0.26 to 1.29)
Australasia	1096 (1060 to 1132)	4.85 (4.69 to 5.01)	1959 (1799 to 2121)	4.21 (3.9 to 4.52)	0.17 (0.08 to 0.26)
Caribbean	728 (666 to 936)	2.46 (2.26 to 3.05)	1634 (1424 to 1901)	3.16 (2.76 to 3.68)	0.67 (0.43 to 0.89)
Central Asia	1725 (1431 to 1967)	2.94 (2.42 to 3.35)	3999 (3464 to 4566)	4.34 (3.77 to 4.92)	0.68 (0.38 to 1.08)
Central Europe	7157 (6848 to 7639)	5.05 (4.83 to 5.4)	10,772 (9820 to 11,748)	5.57 (5.09 to 6.08)	0.63 (0.5 to 0.75)
Central Latin America	2332 (2267 to 2403)	1.97 (1.92 to 2.03)	6167 (5505 to 6892)	2.44 (2.17 to 2.72)	0.72 (0.53 to 0.92)
Central Sub-Saharan Africa	272 (199 to 381)	0.81 (0.58 to 1.05)	786 (526 to 1071)	0.99 (0.65 to 1.35)	0.16 (−0.24 to 0.56)
East Asia	40,077 (29,283 to 50,223)	3.99 (2.94 to 4.96)	70,565 (53,460 to 90,082)	3.59 (2.72 to 4.53)	0.46 (0.13 to 0.86)
Eastern Europe	7941 (7360 to 8419)	3.11 (2.9 to 3.29)	12,717 (11,709 to 13,758)	4.17 (3.86 to 4.5)	0.75 (0.57 to 0.99)
Eastern Sub-Saharan Africa	1653 (1096 to 2427)	1.19 (0.76 to 1.57)	4121 (2903 to 5341)	1.41 (0.94 to 1.77)	0.12 (−0.27 to 0.4)
High-income Asia Pacific	2332 (1967 to 2479)	1.25 (1.05 to 1.33)	4906 (4245 to 5348)	1.51 (1.31 to 1.63)	0.97 (0.76 to 1.17)
High-income North America	14,578 (14,099 to 14,871)	4.46 (4.33 to 4.54)	23,967 (22,466 to 24,781)	4.09 (3.87 to 4.22)	0.25 (0.21 to 0.28)
Oceania	28 (14 to 38)	0.65 (0.34 to 0.88)	71 (37 to 96)	0.69 (0.36 to 0.92)	0.21 (−0.01 to 0.45)
Southeast Asia	6081 (4373 to 7517)	1.82 (1.33 to 2.27)	14,216 (10,394 to 17,094)	2.08 (1.54 to 2.5)	0.56 (0.22 to 0.87)
Southern Latin America	1243 (1130 to 1356)	2.63 (2.39 to 2.87)	2306 (2149 to 2456)	2.83 (2.63 to 3.02)	0.36 (0.21 to 0.53)
Southern Sub-Saharan Africa	498 (383 to 634)	1.46 (1.1 to 1.83)	1231 (901 to 1462)	1.88 (1.36 to 2.22)	0.61 (0.36 to 0.9)
Tropical Latin America	4216 (4007 to 4469)	3.69 (3.51 to 3.94)	12,102 (11,477 to 12,611)	4.8 (4.55 to 5.01)	0.92 (0.8 to 1.05)
Western Europe	21,670 (21,089 to 22,079)	4.33 (4.24 to 4.41)	33,436 (31,382 to 34,905)	4.34 (4.14 to 4.5)	0.36 (0.3 to 0.41)
Western Sub-Saharan Africa	657 (451 to 869)	0.39 (0.28 to 0.49)	2113 (1104 to 2719)	0.56 (0.31 to 0.7)	0.27 (−0.23 to 0.59)

For comparisons of incidence or mortality rates between two time points, the difference is considered statistically significant if the two 95% UIs do not overlap.

Abbreviations: ASMR, age-standardized mortality rates; GBD, Global Burden of Diseases, Injuries, and Risk Factors Study; SDI, socio-demographic index; UI, uncertainty intervals.

#### The decisive impact of socioeconomic development levels

**Figure 2.**
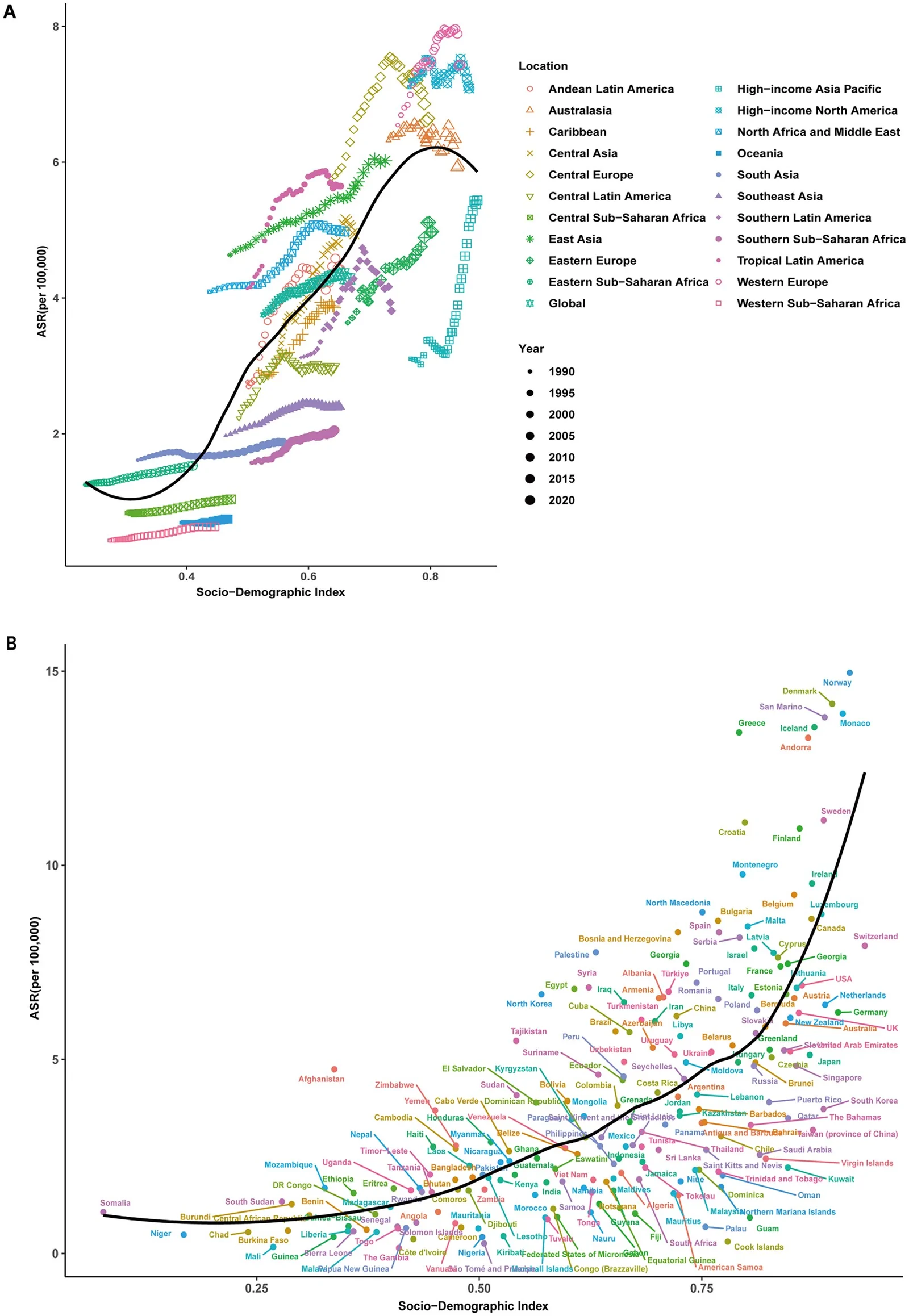
The association between ASR and SDI at the regional (A) and national (B) levels. ASR, age-standardized incidence rate; SDI, socio-demographic index.

We explored the relationship between ASR and SDI. The results were summarized in Figure 2, which showed a significant positive correlation between SDI and ASR at both the regional (Figure 2A) and national (Figure 2B) levels, indicating that regions or countries with higher SDI might have a higher incidence of CNS cancers. As shown in Figure 2A, at the regional level, the ASR levels in Andean Latin America, Central Europe, high-income North America, and Australasia were higher than expected based on the SDI from 1990 to 2021. Nevertheless, it could be observed that the ASRs in these regions experienced a downward trend after the peak. Likewise, the results in Figure 2B illustrated the positive correlation between SDI and ASR at the national level, with several countries having higher ASR levels than expected based on the SDI from 1990 to 2021, such as Norway, Denmark, Monaco, and Iceland.

This burden was distributed unequally. For different SDI regions, the highest ASIR of CNS cancers per 100,000 population in 1990 (5.39; 95% UI, 4.79 to 6.81) and 2021 (6.38; 95% UI, 6.08 to 6.64) occurred in high SDI, which highly correlated with the widespread availability of advanced imaging techniques (such as Magnetic Resonance Imaging) and pathological diagnostic capabilities. The ASMR of CNS cancers per 100,000 population in 1990 (3.80; 95% UI, 3.37 to 4.52) and 2021 (3.94; 95% UI, 3.36 to 4.51) was highest in high-middle SDI. Collectively, the high SDI regions didn’t exhibit the highest ASMR, due to their relatively well-established multidisciplinary treatment system. Conversely, some high-middle SDI regions (eg, Eastern Europe) bore the highest ASMR (eg, Montenegro, 7.77 per 100,000 population), reflecting deficiencies in the chain from diagnosis to effective treatment. Low-SDI regions displayed a pattern of “under-reported incidence and a high mortality burden,” highlighting the dual challenge of insufficient diagnosis and lack of treatment access. Socioeconomic development level, measured by SDI, was a decisive factor shaping this geographical inequality.

#### Trends of the ASR in the future

**Figure 3.**
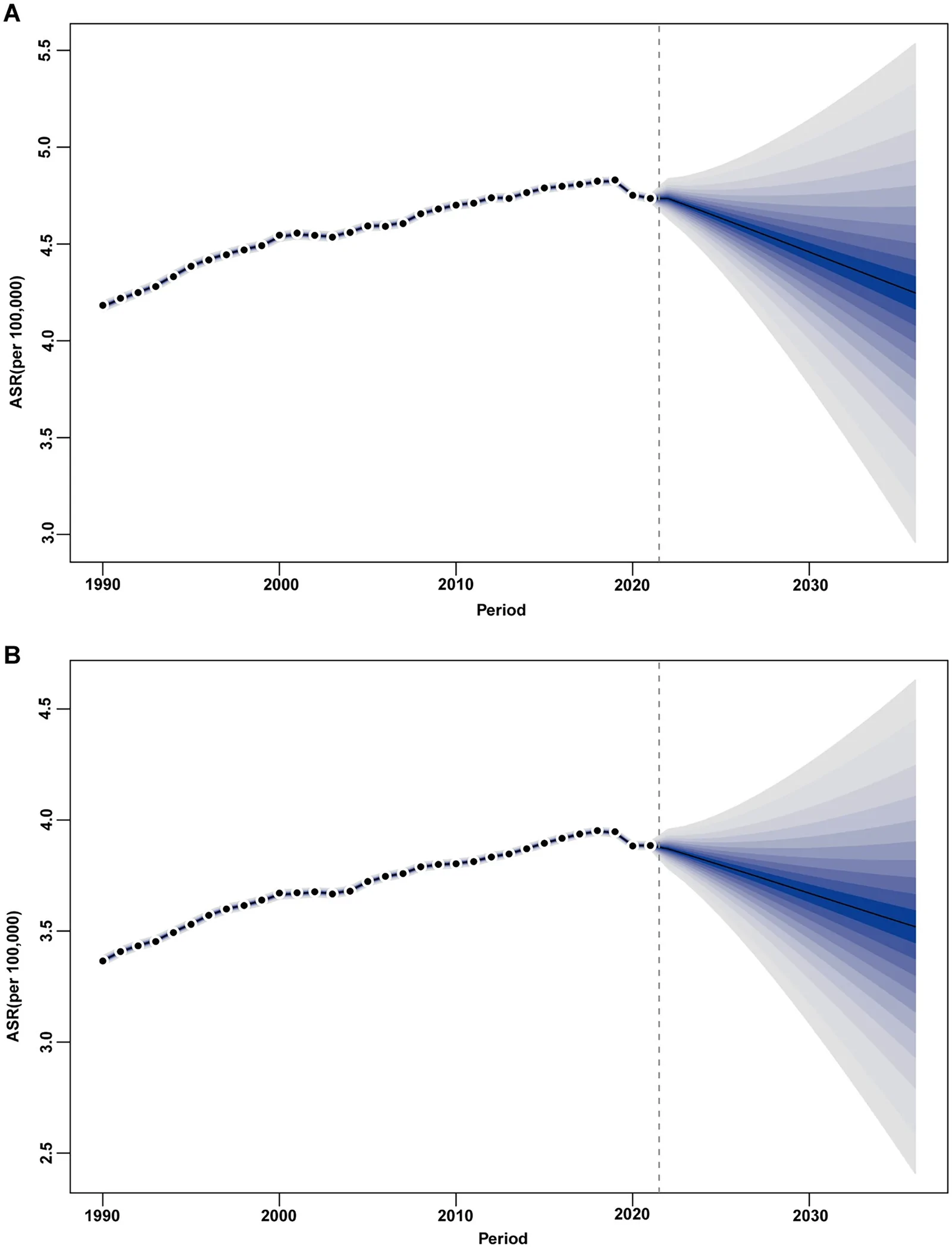
Trends of ASR from 2021 to 2036 in males (A) and females (B) predicted by Bayesian age-period-cohort (BAPC) models. ASR, age-standardized incidence rate.

To understand the trend of ASR of CNS cancers after 2021, we applied Bayesian age-period-cohort models to predict the ASR from 2021 to 2036 by sex, and the results were summarized in Figure 3. As shown in Figure 3A, after 2021, the ASR for males was expected to decrease from 4.74 per 100,000 population in 2021 to 4.25 per 100,000 population in 2036. Also, the predictive results indicated that the ASR for females would decrease annually (Figure 3B), from 3.88 per 100,000 population in 2021 to 3.52 per 100,000 population in 2036. The predictive model indicated that the ASR for males may continue to rise slowly, while the decline curve of ASR for females would remain very gradual. This sent a clear warning that without fundamentally overcoming treatment resistance in CNS cancers, relying solely on improved diagnosis would not significantly reduce the overall burden of death and disability they cause. Macro-level data thereby redirected our focus toward more aggressive tumor subtypes and their therapeutic bottlenecks.

### Meso-Level Perspective: Clinical Heterogeneity and Therapeutic Bottlenecks from SEER

#### Histological subtypes are the root cause of prognostic differences

**Figure 4.**
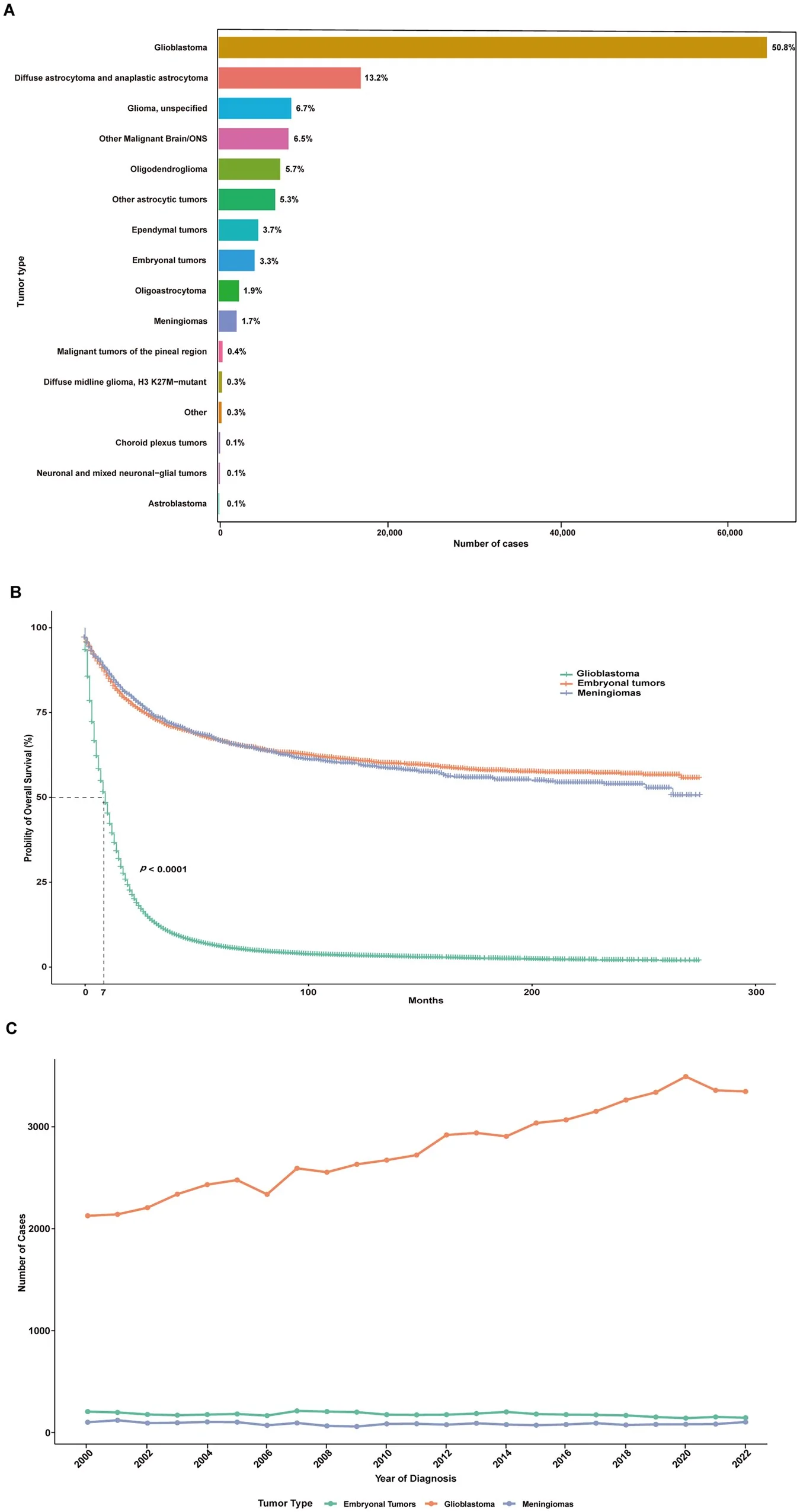
A composite figure with three panels. Figure 4A is a horizontal bar chart showing the relative distribution of brain tumor types, with tumor types listed in descending order of case numbers on the y-axis and case counts on the x-axis, with percentages labeled at the end of each bar. Figure 4B contains Kaplan-Meier survival curves for three brain tumor types, with time since diagnosis in months (0–300 months) on the x-axis and overall survival percentage on the y-axis, demonstrating statistically significant differences among the three groups (*P* < 0.0001). Figure 4C displays annual incidence trends from 2000 to 2022 for three brain tumor types using data from the SEER database, with year of diagnosis on the x-axis and case numbers on the y-axis, showing the overall trend of each tumor type over the study period.

SEER data clearly delineated the divergent fates within CNS cancers in the United States. We selected three representative subtypes of central nervous system tumors: GBM (highly lethal & treatment-challenging), embryonal tumors (intermediate prognosis & management complexity), and meningioma (high diagnostic sensitivity & favorable prognosis/benign nature). Glioblastoma (GBM, WHO grade IV), the most common malignant primary brain tumor (Figure 4A), accounted for over half of all malignant CNS neoplasms, with a median survival of merely 14-16 months^26-29^ and a 5-year survival rate of 10%.^30^ In contrast, meningiomas constituting a significant proportion demonstrated a 5-year overall survival of 66% and an estimated 10-year overall survival of just 14%-24%.^31-34^ Thus, the plateaued mortality in “CNS cancer” mortality trends at the macroscopic level was essentially driven by the dismal prognoses of a few highly aggressive and therapy-resistant subtypes, particularly GBM (Figure 4B and C).

This sent a clear warning: without fundamentally overcoming the therapeutic bottleneck in high-grade CNS cancers, particularly GBM, improved diagnostic capabilities alone would not substantially reduce the overall burden of death and disability caused by these malignancies. SEER data accordingly redirected our focus toward the more aggressive cancer subtype and the underlying treatment challenges.

#### Therapeutic ceiling as the core barrier to survival breakthrough

For newly diagnosed GBM, maximal safe resection followed by concurrent chemoradiotherapy and adjuvant chemotherapy constitutes the standard of care.^27^ SEER data confirmed that while this regimen improved survival, its therapeutic ceiling remained extremely low. Most patients ultimately relapsed, with scarce treatment options available upon recurrence, often leading to poor outcomes. Therefore, there was an urgent need to elucidate the molecular mechanisms underlying therapeutic resistance in GBM, which aims to develop more effective treatment strategies and provide a solid foundation for clinical decision-making.

## Discussion

To effectively develop public health initiatives aimed at preventing and managing CNS cancers, it was crucial to understand the populations at high risk and realize their epidemiological characteristics. For GBD 2021, we analyzed data from cancer registration and vital statistics systems to provide an updated and comprehensive estimation of global, regional, and national levels of CNS cancers’ incidence and mortality from 1990 to 2021, as well as a projection to 2036. First, it focused on the current states of CNS cancers at the global, regional, and national levels. Second, the study systematically described the trends in CNS cancer incidence and mortality rates from the past to the present and for the next 15 years across the full-time continuum in different regions and countries. Third, this study broke through the limitations of previous research that mainly focused on the pathogenesis and treatment options of CNS cancers, and explicitly proposed a theoretical framework that embedded the burden of CNS cancers within the social determinants of health.

Our results indicated that the global burden of CNS cancers increased between 1990 and 2021, which could be evidenced by the increase in incident cases, deaths, and DALYs. Nevertheless, despite the increase in ASIR, ASMR and age-standardized DALYs (Extended Data, Table 1) were decreasing between 1990 and 2021 (although the changes were not significant), possibly due to timely and accurate diagnosis, as well as the improvements in therapeutic regimens. For most regions, the ASIR and ASMR increased as SDI improved, which could be considered that higher SDI regions had more accurate diagnostic measures and more effective treatment modalities, leading to more incident cases and deaths. Our estimates were consistent with other studies in quantifying the global incidence and related deaths of CNS cancers. The GLOBOCAN study estimated 308,102 incident cases and 251,329 deaths in 2020.^35^ The GBD 2021 estimated 351,794 incident cases (95% UI, 304,876-398,842) and 254,652 deaths (95% UI, 219,773-290,545) for the same year. Both GBD and GLOBOCAN also consistently show that there are significant regional differences in the ASIR and ASMR of CNS cancers.

Diseases can have an influence on the nervous system throughout life, such as by disrupting brain growth; impairing cognitive, sensory, socioemotional, and motor function and behavior; and damaging the brain, spinal cord, or peripheral nerves. This group of diverse diseases includes congenital and neurodevelopmental disorders, cerebrovascular and neurodegenerative diseases, nervous system infections, neuromuscular or peripheral nervous system diseases, neuro-immunological diseases, traumatic injuries, and nervous system cancers, collectively referred to as neurological disorders or nervous system conditions, which vary in terms of etiology, symptoms, and course. Some neurological disorders can lead to lifelong disability, while others are associated with high mortality rates; some are treatable or preventable, while others are incurable.

The extension of life expectancy can be considered one of the greatest achievements of health systems worldwide. However, the increase has also led to a rise in age-related neurological diseases, such as CNS cancers, Alzheimer’s disease and other dementias, and stroke, which compels global health policies to focus not only on survival but also on minimizing health loss due to disability by promoting function and independence. Not all neurological burdens are related to population aging,^36^ thus, it was crucial to quantify the overall health loss associated with neurological diseases across the entire life cycle.

In the early stage, there are no obvious and characteristic symptoms indicating the occurrence of CNS cancers. Symptoms such as headaches or epileptic seizures are relatively common and nonspecific. For accurate diagnosis, it is substantial to utilize more advanced diagnostic facilities for confirmation, such as radiological examinations. Perhaps the most significant global health challenge associated with CNS cancers is the demand for highly specialized medical and surgical care for diagnosis and long-term management. Headaches are the most common neurological disorders worldwide, but few headache sufferers have CNS cancers.^37^ The optimal treatment paradigm for CNS cancers requires multidisciplinary cooperation, combining biopsy or surgical resection with postoperative radiotherapy and chemotherapy.^38^ Patients mandate access to intensive care units, neurosurgical, highly specialized radiation, and neuro-oncology services, which are primarily located in urban areas and countries with advanced healthcare systems.^39^^,^^40^ Additionally, the relative rarity of CNS cancers co-occurring with other cancers in adults makes a lower priority in low-resource settings. Consequently, disparities in access to these services are magnified across the entire sociodemographic spectrum. Despite the efforts of GBD to correct for uncertainty, ascertainment bias might partially explain the increase in CNS cancer incidence during this period. Therefore, the influence caused by this bias on the whole increase in ASIR requires further research.

Our analysis indicated that ASMR declined, while ASIR increased, which could be interpreted as improvements in survival rates associated with higher SDI. This result was also in line with the findings of the CONCORD-3 study,^41^ which included pooled data from 37.5 million patients from 71 countries across 15 years. During this time, survival rates for CNS cancers stabilized, but did indeed improve by 3%-10% in several regions with higher SDI, including high-income North America (USA, Canada), western Europe (Iceland, Norway, Sweden, the UK, Denmark, France, Switzerland), and high-income Asia Pacific (South Korea, Singapore). Globally, the ASIR of CNS cancers was increasing, while ASMR and DALYs were decreasing. This relationship became inconsistent for SDI regions. The increasing ASIR for higher quintiles of SDI, but was inverted for low SDI quintiles. Nevertheless, the decreasing ASMR for low SDI quintiles, which was contrary to that of higher SDI quintiles.

These results suggested that DALYs associated with CNS cancers were disproportionately present in areas with lower SDI, which might reflect a lack of highly specialized services needed to treat these intricate diseases. These disparities could lead to delayed diagnoses and an inability to effectively implement treatment that prevents or delays death at least. The heterogeneity observed in CNS cancer incidence may reflect a combination of factors, including genetic predisposition, environmental exposures, and the above-mentioned impacts of access to healthcare. Previous studies have shown that CNS cancers, particularly glioblastoma, were more common in white populations than in Asian or African populations.^42^ Our data also supported this view. The highest incidence rates were observed in Western and Central Europe, and the lowest were in Western Sub-Saharan Africa. There is evidence that this pattern is independent of SDI. For instance, in the regions with the highest SDI, the incidence of CNS cancers and related diseases in Central Europe is more than three times that of the high-income Asia-Pacific region. Extensive genetic susceptibility could make clear the differences in incidence rates among several populations, particularly given that similar SDI regions should have equal access to the diagnostic and therapeutic modalities. However, it was important to note that environmental factors and exposures among these populations may play a significant role. Ionizing radiation is consistently linked to an increased risk, while atopic diseases are consistently associated with a decreased risk, which are the only risk factors that evidence consistently supports.^43-47^

This study revealed that central nervous system tumors continued to pose a severe disease burden, with a much slower decline in mortality compared to many other malignancies, particularly glioblastoma. This epidemiological observation directly points to a core clinical challenge: the current standard treatment regimen combining surgery, radiotherapy, and temozolomide chemotherapy had reached a therapeutic plateau, urgently necessitating breakthrough strategies. One of the key intrinsic factors contributing to this treatment bottleneck was the inherent or acquired therapy resistance (micro-level perspective). Recent basic and translational research has gradually uncovered molecular mechanisms, such as the maintenance of cancer stem cell properties leading to abnormal activation of DNA damage repair pathways,^48^^,^^49^ the immunosuppressive state of the tumor microenvironment,^50^ and specific epigenetic regulation.^51^ These collectively formed a complex network underlying radiotherapy resistance.

It was worth noting that research aimed at elucidating new mechanisms of radiotherapy resistance in GBM is ongoing. Our team is currently investigating the mechanism by which HEG1, through binding to Cx43, stabilizes gap junctions, thereby disrupting copper homeostasis, inhibiting cuproptosis, and leading to radiotherapy resistance in GBM. These data are unpublished. We believed that only through sustained basic research to deeply understand the nature of radio-resistance or novel develop novel combination therapies. Ultimately, translating breakthroughs in these microscopic mechanisms into a significant downward shift in the GBM mortality curve on the macro-epidemiological map represented the ultimate challenge in the field of neuro-oncology and the long-term goal of our research.

The greatest strength of this study lay in its integration of the global perspective from GBD and the high-resolution subtype data from SEER. It should be noted that SEER data originate from the United States, and the absolute incidence rates, healthcare accessibility, and diagnostic standards it reflects may differ from those in other regions, particularly in low- to middle-SDI settings. However, the observed variations in how subtypes respond to advances in diagnostic technology, as well as the persistently high mortality burden of certain aggressive subtypes such as GBM, are underpinned by biological and clinical logic that holds broader relevance. Another strength of GBD estimation is the use of predictive covariates in the estimation. The limitation of the study primarily stemmed from the limited quantity and high heterogeneity of the input data, which our analysis could only overcome partially. Despite our accumulation of a substantial amount of international vital registration, cause-of-death inference, and clinical management data, there is still a lack of primary data available for many regions and years. High-quality initial data is the key to advancing scientific knowledge of CNS cancers, but without modeling, the absence of primary data could be misinterpreted as an absence of burden. Fourth, this study integrated data from GBD and SEER, but their inherent differences warranted careful interpretation. In addition, GBD includes only malignant tumors (ICD-10 codes C70-C72), whereas SEER includes both malignant and non-malignant/borderline tumors. Therefore, we did not directly compare absolute incidence or mortality rates between the two databases. Instead, we adopted a trend-comparison strategy: GBD was used to depict global trends in overall malignant burden, while SEER was used to reveal subtype-level trend patterns. This approach is less sensitive to definitional differences. Moreover, SEER data represent only the US population; its findings cannot be directly generalized globally, especially to low- and middle-income countries with different healthcare resources. In this study, we positioned SEER as a “high-resolution model” to understand mechanisms underlying subtype-specific trends, rather than as a direct global proxy. Future studies incorporating high-quality cancer registry data from Europe, Asia, and other regions are needed to validate the generalizability of our findings.

## Conclusion

In conclusion, the global burden of CNS cancers arises from the complex interplay of diagnostic, therapeutic, and biological factors across multiple scales, a reality that cannot be adequately captured through any single research lens. It was important to acknowledge that current understanding of the global burden relies predominantly on data from regions with established cancer registries (eg, GBD and SEER). In many low- and middle-income countries, the absence of cancer registries, limited access to diagnostics and treatments, and the complex interplay between medicine and culture likely exacerbate the burden. The “macro‑meso‑micro” integrated analytical framework proposed in this study systematically connected GBD epidemiological data, SEER clinical records, and established tumor biology knowledge to form a conceptual system capable of deciphering the evolution of disease burden, identifying pivotal scientific challenges, and guiding translational pathways. The framework further highlighted that the fundamental strategy to alleviate this burden lay in overcoming therapeutic resistance in high‑grade subtypes, epitomized by GBM. Therefore, we advocate following a coordinated pathway of “macro‑level surveillance for target identification—clinical data for problem definition—mechanistic research for breakthrough exploration.” We call upon the global neuro‑oncology community to adopt this integrated approach, strengthen interdisciplinary collaboration, and jointly advance the translation of multi‑scale knowledge into effective clinical interventions, thereby achieving tangible reductions in the burden of disease.

## Data Availability

The data used to support the findings of this study are available from the GBD 2021 and the SEER database.

## References

[1] Price M , Ballard CAP, Benedetti JR, Kruchko C, Barnholtz-Sloan JS, Ostrom QT. CBTRUS statistical report: primary brain and other central nervous system tumors diagnosed in the United States in 2018-2022. Neuro-oncology. 2025;27:iv1-iv66. 10.1093/neuonc/noaf194 41092086 PMC12527013

[2] Ostrom QT , Cioffi G, Gittleman H, et al CBTRUS statistical report: primary brain and other central nervous system tumors diagnosed in the United States in 2012-2016. Neuro-oncology. 2019;21:v1-v100. 10.1093/neuonc/noz150 31675094 PMC6823730

[3] Bray F , Ferlay J, Soerjomataram I, Siegel RL, Torre LA, Jemal A. Global cancer statistics 2018: GLOBOCAN estimates of incidence and mortality worldwide for 36 cancers in 185 countries. CA Cancer J Clin. 2018;68:394-424. 10.3322/caac.21492 30207593

[4] Mitusova K , Peltek OO, Karpov TE, Muslimov AR, Zyuzin MV, Timin AS. Overcoming the blood-brain barrier for the therapy of malignant brain tumor: current status and prospects of drug delivery approaches. J Nanobiotechnol. 2022;20:412. 10.1186/s12951-022-01610-7 PMC9479308 36109754

[5] Howlader N , Cronin KA, Kurian AW, Andridge R. Differences in breast cancer survival by molecular subtypes in the United States. Cancer Epidemiol Biomarkers Prev. 2018;27:619-626. 10.1158/1055-9965.EPI-17-0627 29593010

[6] Park EH , Min SY, Kim Z, et al; Korean Breast Cancer Society. Basic facts of breast cancer in Korea in 2014: the 10-year overall survival progress. J Breast Cancer. 2017;20:1-11. 10.4048/jbc.2017.20.1.1 28382089 PMC5378568

[7] Višnjić A , Kovačević P, Veličkov A, Stojanović M, Mladenović S. Head and neck cutaneous melanoma: 5-year survival analysis in a Serbian university center. World J Surg Oncol. 2020;18:312. 10.1186/s12957-020-02091-4 33250053 PMC7702701

[8] Brunssen A , Jansen L, Eisemann N, et al; GEKID Cancer Survival Working Group. Long-term relative survival from melanoma in Germany 1997-2013. Melanoma Res. 2020;30:386-395. 10.1097/CMR.0000000000000482 30020195

[9] Zhao M , van Straten D, Broekman MLD, Préat V, Schiffelers RM. Nanocarrier-based drug combination therapy for glioblastoma. Theranostics. 2020;10:1355-1372. 10.7150/thno.38147 31938069 PMC6956816

[10] Hemminki K , Försti A, Hansson M. Incidence, mortality and survival in multiple myeloma compared to other hematopoietic neoplasms in Sweden up to year 2016. Sci Rep. 2021;11:17272. 10.1038/s41598-021-96804-8 34446811 PMC8390646

[11] Quaresma M , Coleman MP, Rachet B. 40-year trends in an index of survival for all cancers combined and survival adjusted for age and sex for each cancer in England and Wales, 1971-2011: a population-based study. Lancet (London, England). 2015;385:1206-1218. 10.1016/S0140-6736(14)61396-9 25479696

[12] Leece R , Xu J, Ostrom QT, Chen Y, Kruchko C, Barnholtz-Sloan JS. Global incidence of malignant brain and other central nervous system tumors by histology, 2003-2007. Neuro Oncol. 2017;19:1553-1564. 10.1093/neuonc/nox091 28482030 PMC5737839

[13] Trama A , Botta L, Stiller C, et al; EUROCARE-6 Working Group. Survival of European adolescents and young adults diagnosed with cancer in 2010-2014. Eur J Cancer (Oxford, England: 1990). 2024;202:113558. 10.1016/j.ejca.2024.113558 38489859

[14] Cronin KA , Scott S, Firth AU, et al Annual report to the nation on the status of cancer, part 1: National Cancer Statistics. Cancer. 2022;128:4251-4284. 10.1002/cncr.34479 36301149 PMC10092838

[15] Northcott PA , Robinson GW, Kratz CP, et al Medulloblastoma. Nat Rev Dis Primers. 2019;5:11. 10.1038/s41572-019-0063-6 30765705

[16] Yeo KK , Burgers DE, Brodigan K, et al Adolescent and young adult neuro-oncology: a comprehensive review. Neurooncol Pract. 2021;8:236-246. 10.1093/nop/npab001 34055371 PMC8153805

[17] Surveillance, Epidemiology, and End Results (SEER) Program (www.seer.cancer.gov). SEERStat Database: Incidence—SEER Research Data, 8 Registries, Nov 2025 Sub (1975-2023), National Cancer Institute, DCCPS [online].

[18] Lim SS , Vos T, Flaxman AD, et al A comparative risk assessment of burden of disease and injury attributable to 67 risk factors and risk factor clusters in 21 regions, 1990-2010: a systematic analysis for the Global Burden of Disease Study 2010. Lancet (London, England). 2012;380:2224-2260. 10.1016/S0140-6736(12)61766-8 23245609 PMC4156511

[19] Lozano R , Naghavi M, Foreman K, et al Global and regional mortality from 235 causes of death for 20 age groups in 1990 and 2010: a systematic analysis for the Global Burden of Disease Study 2010. Lancet (London, England). 2012;380:2095-2128. 10.1016/S0140-6736(12)61728-0 23245604 PMC10790329

[20] Murray CJ , Ezzati M, Flaxman AD, et al GBD 2010: design, definitions, and metrics. Lancet (London, England). 2012;380:2063-2066. 10.1016/S0140-6736(12)61899-6 23245602

[21] Global, regional, and national disability-adjusted life-years (DALYs) for 359 diseases and injuries and healthy life expectancy (HALE) for 195 countries and territories, 1990-2017: a systematic analysis for the Global Burden of Disease Study 2017. Lancet (London, England). 2018;392:1859-1922. 30415748 10.1016/S0140-6736(18)32335-3 PMC6252083

[22] Knoll M , Furkel J, Debus J, Abdollahi A, Karch A, Stock C. An R package for an integrated evaluation of statistical approaches to cancer incidence projection. BMC Med Res Methodol. 2020;20:257. 10.1186/s12874-020-01133-5 33059585 PMC7559591

[23] Li S , Chen H, Man J, et al Changing trends in the disease burden of esophageal cancer in China from 1990 to 2017 and its predicted level in 25 years. Cancer Med. 2021;10:1889-1899. 10.1002/cam4.3775 33586344 PMC7940228

[24] Liu N , Yang DW, Wu YX, et al Burden, trends, and risk factors for breast cancer in China from 1990 to 2019 and its predictions until 2034: an up-to-date overview and comparison with those in Japan and South Korea. BMC Cancer. 2022;22:826. 10.1186/s12885-022-09923-4 35906569 PMC9334732

[25] Moher D , Liberati A, Tetzlaff J, Altman DG; PRISMA Group. Preferred reporting items for systematic reviews and meta-analyses: the PRISMA statement. BMJ 2009;339:b2535. 10.1136/bmj.b2535 21603045 PMC3090117

[26] Omuro A , DeAngelis LM. Glioblastoma and other malignant gliomas: a clinical review. JAMA. 2013;310:1842-1850. 10.1001/jama.2013.280319 24193082

[27] Stupp R , Mason WP, van den Bent MJ, et al; National Cancer Institute of Canada Clinical Trials Group. Radiotherapy plus concomitant and adjuvant temozolomide for glioblastoma. N Engl J Med. 2005;352:987-996. 10.1056/NEJMoa043330 15758009

[28] Louis DN , Ohgaki H, Wiestler OD, et al The 2007 WHO classification of tumours of the central nervous system. Acta Neuropathol. 2007;114:97-109. 10.1007/s00401-007-0243-4 17618441 PMC1929165

[29] Chinot OL , Wick W, Mason W, et al Bevacizumab plus radiotherapy-temozolomide for newly diagnosed glioblastoma. N Engl J Med. 2014;370:709-722. 10.1056/NEJMoa1308345 24552318

[30] Stupp R , Hegi ME, Mason WP, et al; National Cancer Institute of Canada Clinical Trials Group. Effects of radiotherapy with concomitant and adjuvant temozolomide versus radiotherapy alone on survival in glioblastoma in a randomised phase III study: 5-year analysis of the EORTC-NCIC trial. Lancet Oncol. 2009;10:459-466. 10.1016/S1470-2045(09)70025-7 19269895

[31] Ostrom QT , Price M, Neff C, et al CBTRUS statistical report: primary brain and other Central nervous system tumors diagnosed in the United States in 2016-2020. Neuro-oncology, 2023;25:iv1-iv99. 10.1093/neuonc/noad149 37793125 PMC10550277

[32] Walsh KM , Price M, Neff C, et al The joint impacts of sex and race/ethnicity on incidence of grade 1 versus grades 2-3 meningioma across the lifespan. Neuro-Oncol Adv. 2023;5:i5-i12. 10.1093/noajnl/vdad020 PMC10243865 37287573

[33] Maier AD , Mirian C, Haslund-Vinding J, et al Granular clinical history and outcome in 51 patients with primary and secondary malignant meningioma. J Neurosurg. 2022;137:1347-1357. 10.3171/2022.1.JNS212723 35276654

[34] Tosefsky K , Rebchuk AD, Wang JZ, et al Grade 3 meningioma survival and recurrence outcomes in an international multicenter cohort. J Neurosurg. 2024;140:393-403. 10.3171/2023.6.JNS23465 37877968

[35] Sung H , Ferlay J, Siegel RL, et al Global cancer statistics 2020: GLOBOCAN estimates of incidence and mortality worldwide for 36 cancers in 185 countries. CA Cancer J Clin. 2021;71:209-249. 10.3322/caac.21660 33538338

[36] Global, regional, and national burden of neurological disorders, 1990-2016: a systematic analysis for the Global Burden of Disease Study 2016. Lancet Neurol. 2019;18:459-480. 30879893 10.1016/S1474-4422(18)30499-X PMC6459001

[37] Global, regional, and national burden of neurological disorders during 1990-2015: a systematic analysis for the Global Burden of Disease Study 2015. Lancet Neurol. 2017;16:877-897. 28931491 10.1016/S1474-4422(17)30299-5 PMC5641502

[38] Preusser M , Marosi C. Neuro-oncology in 2016: advances in brain tumour classification and therapy. Nat Rev Neurol. 2017;13:71-72. 10.1038/nrneurol.2017.3 28106065

[39] Park KB , Johnson WD, Dempsey RJ. Global neurosurgery: the unmet need. World Neurosurg. 2016;88:32-35. 10.1016/j.wneu.2015.12.048 26732963

[40] Bergen DC , Silberberg D. Nervous system disorders: a global epidemic. Arch Neurol. 2002;59:1194-1196. 10.1001/archneur.59.7.1194 12117370

[41] Allemani C , Matsuda T, Di Carlo V, et al; CONCORD Working Group. Global surveillance of trends in cancer survival 2000-14 (CONCORD-3): analysis of individual records for 37 513 025 patients diagnosed with one of 18 cancers from 322 population-based registries in 71 countries. Lancet (London, England). 2018;391:1023-1075. 10.1016/S0140-6736(17)33326-3 29395269 PMC5879496

[42] Chen P , Aldape K, Wiencke JK, et al Ethnicity delineates different genetic pathways in malignant glioma. Cancer Res. 2001;61:3949-3954. 11358811

[43] Andersen ZJ , Pedersen M, Weinmayr G, et al Long-term exposure to ambient air pollution and incidence of brain tumor: the European Study of Cohorts for Air Pollution Effects (ESCAPE). Neuro Oncol. 2018;20:420-432. 10.1093/neuonc/nox163 29016987 PMC5817954

[44] Amirian ES , Zhou R, Wrensch MR, et al Approaching a scientific consensus on the association between allergies and glioma risk: a report from the glioma international case-control study. Cancer Epidemiol Biomarkers Prev. 2016;25:282-290. 10.1158/1055-9965.EPI-15-0847 26908595 PMC4874516

[45] Pearce MS , Salotti JA, Little MP, et al Radiation exposure from CT scans in childhood and subsequent risk of leukaemia and brain tumours: a retrospective cohort study. Lancet (London, England). 2012;380:499-505. 10.1016/S0140-6736(12)60815-0 22681860 PMC3418594

[46] Taylor AJ , Little MP, Winter DL, et al Population-based risks of CNS tumors in survivors of childhood cancer: the British childhood cancer survivor study. J Clin Oncol. 2010;28:5287-5293. 10.1200/JCO.2009.27.0090 21079138 PMC4809645

[47] Mathews JD , Forsythe AV, Brady Z, et al Cancer risk in 680,000 people exposed to computed tomography scans in childhood or adolescence: data linkage study of 11 million Australians. BMJ 2013;346:f2360. 10.1136/bmj.f2360 23694687 PMC3660619

[48] Singh SK , Hawkins C, Clarke ID, et al Identification of human brain tumour initiating cells. Nature. 2004;432:396-401. 10.1038/nature03128 15549107

[49] Bao S , Wu Q, McLendon RE, et al Glioma stem cells promote radioresistance by preferential activation of the DNA damage response. Nature. 2006;444:756-760. 10.1038/nature05236 17051156

[50] Sampson JH , Gunn MD, Fecci PE, Ashley DM. Brain immunology and immunotherapy in brain tumours. Nat Rev Cancer. 2020;20:12-25. 10.1038/s41568-019-0224-7 31806885 PMC7327710

[51] Matesanz-Sánchez R , Peitzsch M, Lange I, et al A novel role of exostosin glycosyltransferase 2 (EXT2) in glioblastoma cell metabolism, radiosensitivity and ferroptosis. Cell Death Differ. 2025;32:1664-1678. 10.1038/s41418-025-01503-w 40234611 PMC12432244

